# Impact of Central vs. Noncentral Predominant Jet Location on Clinical Outcomes: Results From the EXPANDed Studies

**DOI:** 10.1016/j.shj.2025.100743

**Published:** 2025-10-25

**Authors:** Jason H. Rogers, Matthew J. Price, Gagan D. Singh, Paul Mahoney, Mathew Williams, Paolo Denti, Anita Asgar, Janani Aiyer, Rong Huang, Jose Luis Zamorano, Federico M. Asch, Francesco Maisano, Saibal Kar, Ralph Stephan von Bardeleben, Evelio Rodriguez

**Affiliations:** a Division of Cardiovascular Medicine, University of California – Davis, Sacramento, California, USA; b Division of Cardiovascular Diseases, Scripps Clinic, La Jolla, California, USA; c Structural Heart and Complex High Risk Intervention, Sentara, Norfolk, Virginia, USA; d Interventional Cardiology, The Brody School of Medicine at East Carolina University, Greenville, North Carolina, USA; e Cardiac Surgery and Interventional Cardiology, NYU Langone Health, New York, New York, USA; f Department of Transcatheter Therapy of Structural Heart Disease, Department of Cardiac Surgery, San Raffaele University Hospital, Milan, Italy; g Bluhm Cardiovascular Institute of Northwestern, Northwestern Medicine, Chicago, Illinois, USA; h Transcatheter Valve Therapy Research, Montreal Heart Institute, Montreal, Canada; i Abbott Structural Heart, Santa Clara, California, USA; j Transcatheter Valve Therapy Research, Hospital Ramon y Cajal, Madrid, Spain; k Echocardiography Core Lab, MedStar Health Research Institute, Washington, District of Columbia, USA; l Los Robles Regional Medical Center, HCA Healthcare, Thousand, Oaks, California, USA; m Department of Structural Interventions, University Medical Center of Johannes Gutenberg University, Mainz, Germany; n Cardiac Surgery, Ascension Saint Thomas, Nashville, Tennessee, USA

**Keywords:** Heart failure, MitraClip, Mitral transcatheter edge-to-edge repair (M-TEER), Mitral valve repair

## Abstract

**Background:**

Mitral transcatheter edge-to-edge repair (M-TEER) is a treatment option for patients with severe mitral regurgitation (MR) at a high surgical risk. Although most MR involves central A2P2 jets, a subset present with noncentral jets, which may introduce procedural complexity and influence outcomes. The objective of the study was to evaluate the impact of main MR jet location (central [A2P2] versus noncentral [A1/P1 or A3/P3]) on procedural success and clinical outcomes following M-TEER.

**Methods:**

This analysis used the EXPANDed data set, which included patients undergoing M-TEER with MitraClip G3/G4 systems and echocardiographic core laboratory-assessed main MR jet location. One-year clinical, echocardiographic, and functional outcomes were assessed.

**Results:**

A total of 1785 patients had main jets at A2P2 and 81 at A1P1 or A3P3 (non-A2P2). Non-A2P2 patients more frequently had degenerative MR, prior mitral valve procedures, and better left ventricular function. Procedural success was high and comparable (A2P2: 95.9%, non-A2P2: 92.5%; *p* = 0.15), with low 30-day major adverse event rates in both (A2P2: 4.2%, non-A2P2: 7.4%; *p* = 0.16). MR ≤ 1+ was achieved in both groups at 1 year (A2P2: 91%, non-A2P2: 84%, *p* = 0.11). New York Heart Association class ≤ II improved through 1 year in both groups (A2P2: 81%, non-A2P2: 88%). Kansas City Cardiomyopathy Questionnaire overall summary improved significantly with no difference between groups at 1 year (A2P2: Δ13 points, non-A2P2: Δ20 points). One-year all-cause mortality was similar (10.7 vs. 13.7%; *p* = 0.47).

**Conclusions:**

In this largest analysis to date of patients with severe MR, main MR jet location did not affect the safety or effectiveness of the MitraClip system. These findings support the use of M-TEER across a range of anatomical presentations, including non-A2P2 MR jets.

## Highlights

• The impact of main mitral regurgitation jet location on outcomes following MitraClip was evaluated.
• Most patients in EXPANDed had central (95.6%) vs. noncentral jets (4.3%).
• High rates of procedural success regardless of jet location.
• Significant improvement in mitral regurgitation and clinical outcomes regardless of jet location.
• Findings suggest that noncentral jets need not preclude MitraClip treatment.

## Introduction

Mitral regurgitation (MR) remains one of the most prevalent valvular heart diseases globally, contributing substantially to patient morbidity and mortality.^1^ Transcatheter mitral valve (MV) repair, particularly mitral transcatheter edge-to-edge repair (M-TEER) using the MitraClip system, has transformed the therapeutic landscape for patients with severe MR who are at an elevated surgical risk or deemed inoperable.^2^^,^^3^

In clinical practice, the majority of MR cases involve a central regurgitant jet originating from the A2P2 segment of the MV, corresponding to the middle scallop of the posterior leaflet and associated central portion of the anterior leaflet. This anatomical pattern has guided procedural strategies and device design, forming the foundation of most M-TEER experience.^4^ However, a clinically relevant subset of patients presents with noncentral MR jets, arising from lateral (A1/P1) or medial (A3/P3) leaflet segments. Since noncentral MR jets are typically associated with higher chordal density and increased variability in terms of coaptation planes, these anatomies introduce procedural complexity and may influence device positioning, leaflet grasping, and overall repair durability.

The EXPAND and EXPAND G4 trials have shown the safety and efficacy of M-TEER across diverse anatomical presentations.^5^ These studies demonstrated that with iterative device enhancements and growing operator expertise, significant MR reduction is achievable even in anatomically challenging cases. However, the specific impact of the predominant, main MR jet location, central versus noncentral, on procedural success, acute and long-term clinical endpoints remains insufficiently characterized.

This analysis leverages data from the EXPANDed studies to systematically evaluate the influence of main jet location on M-TEER outcomes. By comparing patients with central A2P2 jets to those with non-A2P2 jets, we aimed to characterize the impact of anatomical jet location on outcomes.

## Methods

### Study Design

This analysis used the EXPANDed data set, a pooled, patient-level cohort comprising individuals who underwent M-TEER with the MitraClip G3 or G4 systems as part of the EXPAND (NCT03502811) and EXPAND G4 (NCT04177394) studies. Both studies were designed to assess the real-world safety and effectiveness of the MitraClip systems across a broad spectrum of MR anatomies and patient profiles. The third-generation MitraClip G3 System, or MitraClip NTR/XTR, includes an implant with longer clip arms (12 mm [XTR] vs. 9 mm [NTR]) and delivery system improvements over prior device iterations. The fourth-generation MitraClip G4 System builds on the previous NTR/XTR system with the addition of wider clip sizes (NTW and XTW), independent grasping, and improved clip deployment.

The studies were conducted at 91 sites across 12 countries, including the United States, Canada, several European nations, Saudi Arabia, Israel, and Japan. Patients were enrolled and treated according to regional indications for use, with eligibility determined by site-reported assessments and multidisciplinary heart team evaluations. Enrollment occurred between 2018 and 2019 for EXPAND (1041 patients) and 2020 and 2022 for EXPAND G4 (1164 patients).

### Analysis Population

This analysis evaluated clinical outcomes based on the location of the main MR jet, comparing patients with central A2P2 jets to those with noncentral jets originating outside the A2P2 region. Of the 2205 patients enrolled in the EXPANDed cohort who received a MitraClip implant, 1866 had echocardiographic core laboratory (ECL)-assessed main MR jet location and were included in the final study population. Main jet location was defined as the location of the dominant jet contributing to the severity of mitral regurgitation; a patient could only have 1 location defined, thus patients were categorized as either central (A2P2 group) or noncentral (non-A2P2 group), with the latter encompassing jets from lateral (A1/P1) or medial (A3/P3) leaflet segments. Patients with missing data or nonevaluable origin of jet location were excluded from the analysis.

### Clinical Outcomes and Echocardiographic Assessments

Procedural metrics included index hospitalization length of stay, procedure duration, total device time, number of clips implanted per patient, and acute procedural success. Acute procedural success was defined as MR reduction to ≤2+ without death or the need for surgical MV replacement during the index hospitalization. Safety outcomes included major adverse events (MAEs), defined as a composite of death, myocardial infarction, stroke, surgical MV replacement, single leaflet device attachment (SLDA), leaflet damage, and device embolization.

Additional clinical outcomes included 1-year all-cause mortality, heart failure hospitalization (HFH), functional status assessed by New York Heart Association (NYHA) class, and quality of life measured by the Kansas City Cardiomyopathy Questionnaire overall summary (KCCQ-OS) score.

All transthoracic and transesophageal echocardiograms obtained at baseline, discharge, 30 days, and 1 year were evaluated by 2 independent core laboratories, as previously described.^5^^,^^6^ Leaflet-related adverse events including SLDA, device embolization, and leaflet damage were assessed by the ECL (MedStar Health Research Institute, Columbia, Maryland). MR severity was assessed per the standardized criteria.^7^^,^^8^ Clinical events were adjudicated by an independent clinical events committee for EXPAND and was reported by sites for EXPAND G4. Acute procedural success was defined as MR reduction to ≤2+ without death or surgical MV replacement.

### Statistical Analysis

Continuous variables were summarized as mean ± SD, whereas categorical variables were reported as absolute counts and percentages. Statistical comparisons were performed to assess differences in baseline characteristics, procedural metrics, MAE rates, and MR reduction outcomes at 30-day and 1-year follow-up between A2P2 and non-A2P2 groups. Comparisons were reported using Student’s t-test, Mann-Whitney U test, chi-square test, Fisher exact test, or McNemar’s test depending on the type of outcome. MAEs and device-related complications occurring within 1 year of the index procedure were analyzed in patients who experienced events or remained in the study through the lower bound of the visit window. Time-to-event outcomes, including all-cause mortality and HFH, were estimated using the Kaplan–Meier method, with group comparisons performed using log-rank tests. Patients were censored at their last known event-free date. Multivariable Cox-proportional hazards models were used to assess the association between main jet location and all-cause mortality, HFH, and the composite of both and were adjusted for the following baseline characteristics: left ventricular (LV) ejection fraction (LVEF), LV end-diastolic dimension, LV end-systolic diameter, LV end-systolic volume (LVESV), LV end-diastolic volume, etiology of secondary MR (SMR), prior MR procedure, renal failure, and prior HFH. A 2-sided *p* value < 0.05 was considered statistically significant. All statistical analyses were conducted using SAS (version 9.4; SAS Institute Inc., Cary, NC).

## Results

### Analysis Population and Baseline Characteristics

**Figure 1:**
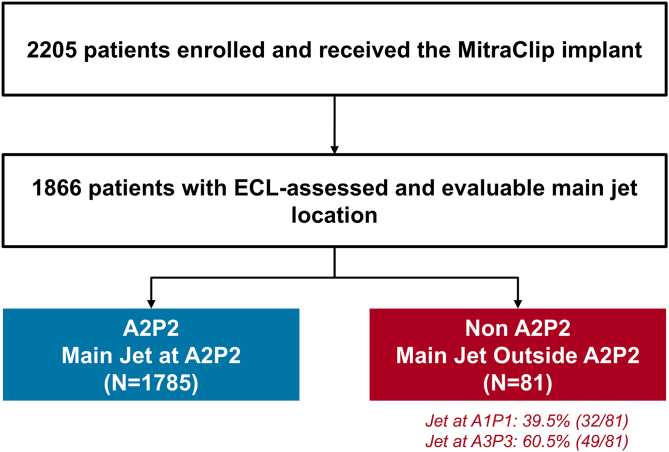
**Analysis flowchart.** Patients with identifiable main mitral regurgitant jet location per echocardiographic core laboratory (ECL) as either A2P2 or non-A2P2 were included.

Of the 2205 patients in the EXPANDed cohort who underwent MitraClip implantation, 1866 had ECL-assessed and evaluable main MR jet location and were included in this analysis. Among these, 1785 patients had a primary jet located at the A2P2 region (A2P2 group), whereas 81 patients had jets originating outside the A2P2 region (non-A2P2 group). Within the non-A2P2 group, 32 had jets at the lateral A1P1 segment and 49 at the medial A3P3 segment (Figure 1).

Baseline demographics of age and sex distribution showed no significant differences between A2P2 and non-A2P2 groups (Table 1). Patients in the non-A2P2 group were more likely to have degenerative MR (68.4 vs. 45.9%; *p* = 0.0002) and a history of prior (MV procedures (17.3 vs. 3.9%; *p* < 0.0001). Of the 70 patients in the A2P2 group with a prior MV procedure, 25 had a prior surgical MV repair, 16 had a surgical annuloplasty ring, and 38 had a mitral transcatheter intervention (including 30 leaflet clips, 1 direct annuloplasty intervention, and 7 unspecified). Of 14 patients in the non-A2P2 group with a prior MV procedure, 3 had a prior surgical MV repair, 3 had a surgical annuloplasty ring, and 10 had a mitral transcatheter intervention (including 9 leaflet clips and 1 direct annuloplasty intervention). The A2P2 group also had a higher prevalence of renal failure (31.9 vs. 21.0%; *p* = 0.04) and prior HFH within 1 year (48.5 vs. 36.1%; *p* = 0.04). LV function was significantly better in the non-A2P2 group, with a higher mean LVEF (LVEF: 57.5% ± 14.1% vs. 49.8% ± 16.1%; *p* = 0.0002) and smaller LVESV (LVESV: 59.6 ± 37.0 mL vs. 78.5 ± 58.3 mL; *p* = 0.02). LV end-systolic diameter was also lower in the non-A2P2 group (3.88 ± 1.08 cm vs. 4.18 ± 1.19 cm; *p* = 0.03).

**Table 1:** Baseline characteristics by main regurgitant jet location

	A2P2 (N = 1785)	Non-A2P2 (N = 81)	*p* value
Age (y)	77.4 ± 9.4 (1785)	77.3 ± 10.0 (81)	0.89
Female	44.5% (794)	46.9% (38)	0.67
CRT/CRT-D/ICD/permanent pacemaker	10.7% (191)	7.4% (6)	0.35
STS repair score (%)	6.2 ± 6.5 (1172)	5.2 ± 4.5 (52)	0.56
STS replacement score (%)	7.9 ± 6.6 (1026)	7.0 ± 5.2 (52)	0.56
Prior mitral valve procedure	3.9% (70)	17.3% (14)	<0.0001
Renal failure	31.9% (566)	21.0% (17)	0.04
Prior HFH within 1 y	48.5% (799)	36.1% (26)	0.04
Degenerative MR	45.9% (739)	68.4% (51)	0.0002
LVEF (%)	49.8 ± 16.1 (1346)	57.5 ± 14.1 (58)	0.0002
LVESV (mL)	78.5 ± 58.3 (1348)	59.6 ± 37.0 (59)	0.02
LVESD (cm)	4.2 ± 1.2 (1569)	3.9 ± 1.1 (69)	0.03
AP diastolic annular dimension (cm)	3.4 ± 0.5 (1509)	3.3 ± 0.5 (63)	0.12

Data presented as mean ± SD or % (n).

*p* value based on Mann-Whitney U test, t-test, or chi-square test.

Abbreviations: AP = anterior-posterior, CRT = cardiac resynchronization therapy, CRT-D = CRT-defibrillator, HFH = heart failure hospitalization, ICD = implantable cardioverter-defibrillator, LV = left ventricle, LVEF = LV ejection fraction, LVESV = LV end-systolic volume, LVESD = LV end-systolic dimension, MR = mitral regurgitation; STS = Society of Thoracic Surgeons.

### Procedural and Safety Outcomes

Procedural metrics were comparable between the A2P2 and non-A2P2 groups, despite differences in anatomical jet location (Table 2). The mean index hospital length of stay was 5.1 ± 8.7 days in the A2P2 group and 5.8 ± 10.6 days in the non-A2P2 group (*p* = 0.56). Procedure duration (86.9 ± 45.7 vs. 82.7 ± 35.8 minutes; *p* = 0.31), total device time (49.0 ± 38.8 vs. 47.0 ± 33.0 minutes; *p* = 0.65), and the number of clips implanted per patient (1.4 ± 0.6 in both groups; *p* = 0.76) were also similar. The proportions of 1-clip (A2P2: 60.2%, non-A2P2: 63.0%) and 2-clip cases (A2P2: 35.8%, non-A2P2: 32.1%) were similar between groups. Acute procedural success was high in both groups (A2P2: 95.9% vs. non-A2P2: 92.5%; *p* = 0.15).

**Table 2:** Procedural characteristics by main regurgitant jet location

	A2P2 (N = 1785)	Non-A2P2 (N = 81)	*p* value
Acute procedural success (%)	95.9% (1707)	92.5% (74)	0.15
Procedure duration (minutes)	86.9 ± 45.7 (1785)	82.7 ± 35.8 (81)	0.31
Total device time (minutes)	49.0 ± 38.8 (1771)	47.0 ± 33.0 (81)	0.65
Number of clips implanted per patient	1.4 ± 0.6 (1785)	1.4 ± 0.6 (81)	0.76
1 clips	60.2% (1075)	63.0% (51)	-
2 clips	35.8% (639)	32.1% (26)	-
3 clips	3.8% (68)	4.9% (4)	-
4 clips	0.1% (2)	-	-
5 clips	0.1% (1)	-	-
Index procedure length of stay in hospital (d)	5.1 ± 8.7 (1785)	5.8 ± 10.6 (81)	0.56

Data presented as % (n), or mean ± SD (n). *p* value based on t-test or chi-square test.

**Figure 2:**
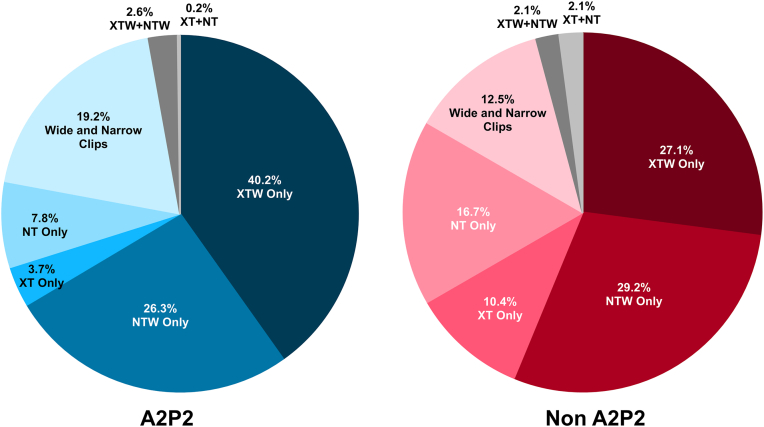
MitraClip G4 Clip usage in patients with A2P2 or non-A2P2 main mitral regurgitant jets.

Clip usage by jet location, limited to MitraClip G4 devices, is shown in Figure 2. In the non-A2P2 group, NTW was the most frequently used clip, contrasting with XTW, which was the predominant choice in the A2P2 group. Notably, nonwide clips (XT and NT) were used more often in non-A2P2 cases (27.1%) compared to A2P2 (11.4%), reflecting a shift in strategy based on anatomical considerations.

**Figure 3:**
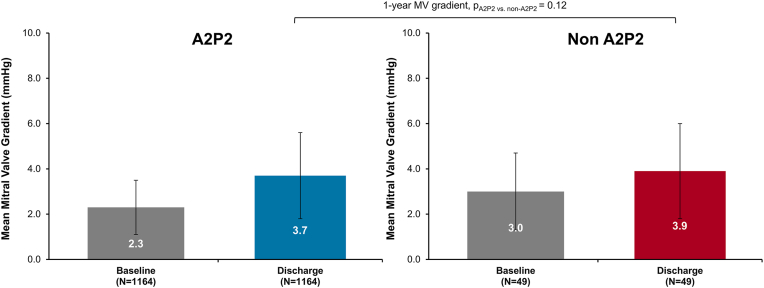
**Mean mitral valve gradient through discharge for all eligible patients.** Mean mitral gradient through discharge for full cohort by main jet location. Paired analysis shown. Abbreviation: MV, mitral valve.

The mean MV gradient assessed by the ECL and paired between baseline and discharge is shown in Figure 3. After M-TEER, the discharge mean gradient was similar and below the clinically significant threshold of 5 mmHg regardless of main jet location (A2P2: 3.7 mmHg, non-A2P2: 3.9 mmHg, *p* = 0.12).

The overall 30-day MAE rate was low and similar across both groups. MAEs occurred in 4.2% of patients in the A2P2 group and 7.4% in the non-A2P2 group (*p* = 0.16). Rates of individual events including death (2.0 vs. 2.5%), MV replacement (0.9 vs. 1.2%), and SLDA (1.1 vs. 2.5%) were similar between groups. These findings suggest that despite the potentially greater anatomical complexity in the non-A2P2 group, procedural safety was comparable between groups (Table 3). The 30-day chordal entrapment rates were low and similar A2P2 and non-A2P2 groups (A2P2: 0.1%, non-A2P2: 0.1%, *p* = 0.82).

**Table 3:** Major adverse events through 30 days by main regurgitant jet location

	A2P2 (N = 1785)	Non-A2P2 (N = 81)	*p* value
Overall major adverse events	4.2% (75)	7.4% (6)	0.16
All-cause mortality	1.0% (35)	2.5% (2)	0.67
Myocardial infarction	0.1% (2)	0.0% (0)	0.91
Stroke	0.7% (13)	1.2% (1)	0.47
Mitral valve replacement	0.9% (16)	1.2% (1)	0.53
Single leaflet device attachment	1.1% (19)	2.5% (2)	0.23
Embolization	0.1% (1)	0.0% (0)	0.96

Data presented as % (n).

*p* value based on chi-square test.

### Echocardiographic Outcomes

**Figure 4:**
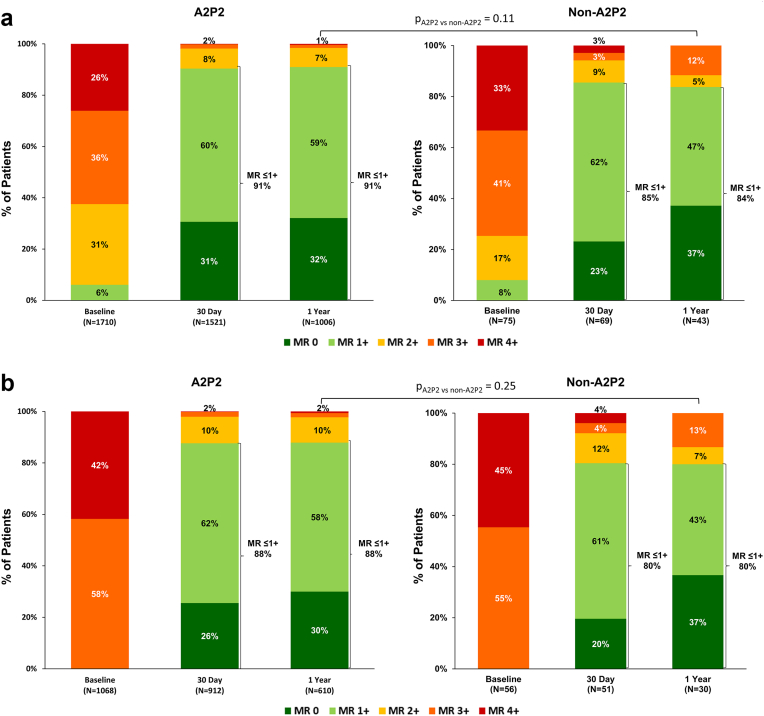
**Mitral regurgitation through 1 year for all eligible patients.** Mitral regurgitation (MR) severity through 1 year for (A) full cohort by main MR jet location and (B) in patients with baseline MR of 3+/4+ only. Unpaired analysis shown.

Both groups experienced substantial MR reduction through 1 year (Figure 4A). At baseline, 6% of A2P2 patients and 8% of non-A2P2 patients had MR ≤ 1+. At 30 days, MR ≤1+ was achieved in 91% of A2P2 patients and 85% of non-A2P2 patients (p_A2P2 vs non-A2P2_ = 0.19). At 1 year, MR ≤ 1+ was sustained in 91% of A2P2 patients and 84% of non-A2P2 patients (p _A2P2 vs non-A2P2_ = 0.11), indicating durable MR reduction regardless of main MR jet location. Consistent with the full cohort results, reduction of MR severity for patients with only severe MR (MR ≥ 3+) at baseline was comparable between A2P2 and non-A2P2 groups (Figure 4B).

**Figure 5:**
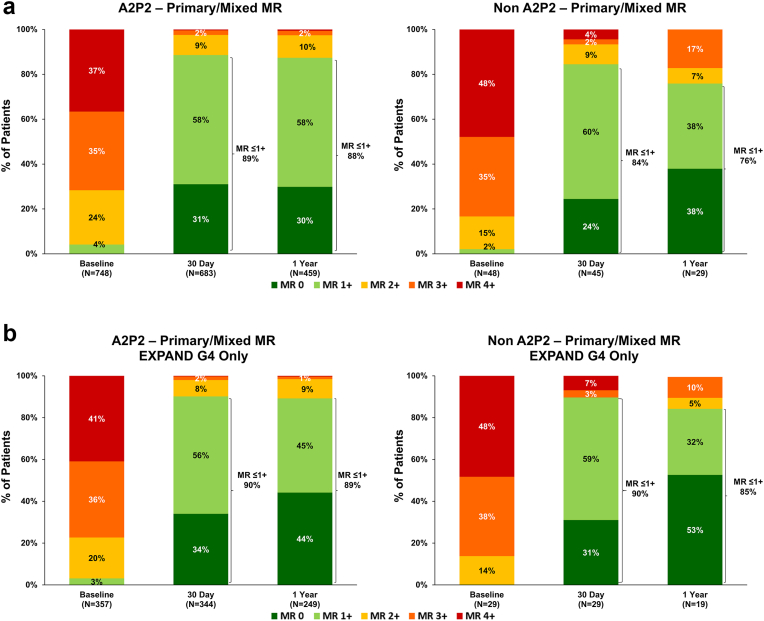
**Mitral regurgitation through 1 year in patients with primary or mixed etiology MR.** MR severity through 1 year for (A) all patients with primary and mixed MR or (B) only patients treated with MitraClip G4 in EXPAND G4 by main MR jet location. Unpaired analysis shown.

**Figure 6:**
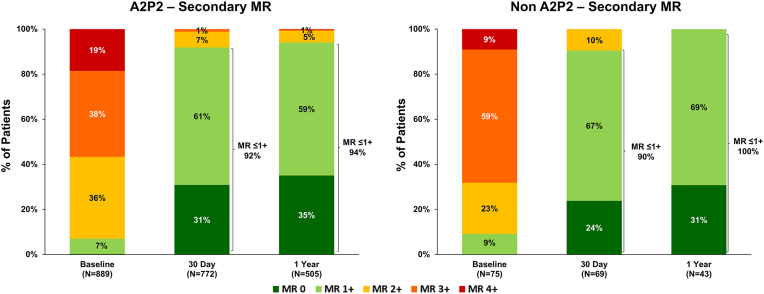
**Mitral regurgitation (MR) through 1 year in patients with secondary MR.** MR severity through 1 year for patients with secondary MR by main MR jet location. Unpaired analysis shown.

Patients with primary MR (PMR) experienced substantial MR reduction regardless of their main MR jet location (Figure 5A). In the A2P2 group, 4% had MR ≤ 1+ at baseline, increasing to 89% at 30 days and 88% at 1 year. In the non-A2P2 group, MR ≤ 1+ was observed in 2% at baseline, which improved to 84% at 30 days and 74% at 1 year. To explore whether device iteration could influence outcomes in the non-A2P2 group, we performed a sub analysis limited to patients with PMR treated with MitraClip G4 devices in both groups. In this subset, patients with PMR in the non-A2P2 group showed 1-year MR reduction more comparable to those in the A2P2 group (PMR, MR ≤1+: A2P2: 89%, non-A2P2: 85%, Figure 5B). Although sample sizes were limited (non-A2P2: N = 29 at 30 days, N = 19 at 1 year), the data suggest that the use of MitraClip G4 devices may help achieve similar MR ≤ 1+ rates even in patients with jets outside of A2P2. Patients with SMR also experienced significant MR reduction (Figure 6). In the A2P2 SMR group, MR ≤ 1+ improved from 7% at baseline to 92% at 30 days and 94% at 1 year. In the non-A2P2 SMR group, MR ≤ 1+ rose from 9% at baseline to 90% at 30 days and reached 100% at 1 year, indicating elimination of significant MR.

### Functional and Quality-Of-Life Improvements

**Figure 7:**
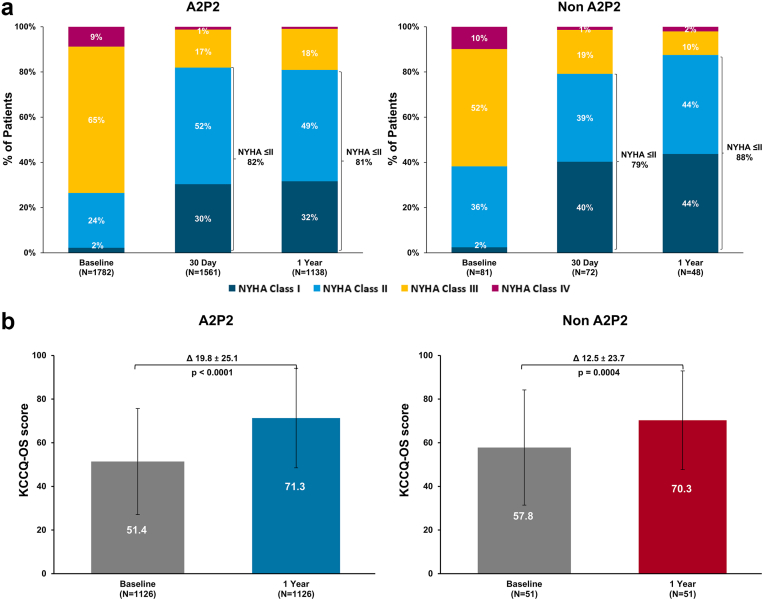
**Functional capacity and quality-of-life improvement through 1 year.** (A) New York Heart Association (NYHA) class through 1 year by main jet location and (B, paired analysis) quality of life, as assessed by Kansas City Cardiomyopathy Questionnaire Overall Summary (KCCQ-OS) score for patients by main jet location.

Functional status, measured by NYHA class, improved substantially through 1 year in both groups (Figure 7A). In the A2P2 group, the proportion of patients with NYHA class ≤ II increased from 26% at baseline to 82% at 30 days and remained stable at 81% at 1 year. Similarly, in the non-A2P2 group, NYHA class ≤ II improved from 38% at baseline to 79% at 30 days and 88% at 1 year. Differences between groups at both time points were not statistically significant, indicating comparable functional improvement regardless of main MR jet location.

Both groups experienced significant improvements in KCCQ-OS scores from baseline to 1 year (Figure 7B). The A2P2 group showed a mean increase of 20 points (*p* < 0.0001 compared to baseline), whereas the non-A2P2 group improved by 13 points (*p* = 0.0004 compared to baseline). Although the magnitude of improvement was greater in the A2P2 group, potentially attributed to lower baseline KCCQ-OS scores, the difference between groups at 1 year was not statistically significant.

### Clinical Outcomes

**Figure 8:**
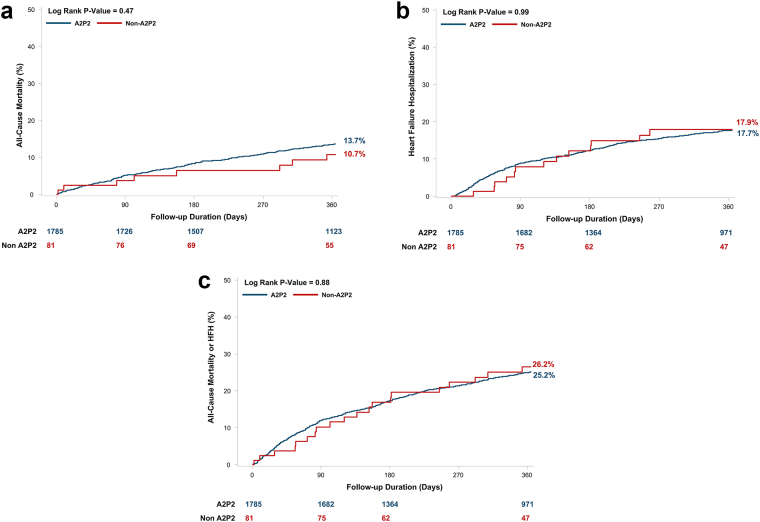
**All-cause mortality and heart failure hospitalization (HFH) though 1 year.** (A) Kaplan-Meier all-cause mortality, (B) HFH, and (C) all-cause mortality/HFH were not significantly different between patients by main jet location through 1 year.

At 1 year, all-cause mortality was 13.7% in the A2P2 group and 10.7% in the non-A2P2 group (Figure 8A). There was no statistically significant difference between groups (log-rank *p* = 0.47), suggesting similar survival outcomes regardless of main MR jet location. This remained nonsignificant after adjustment in Cox-proportional hazards analysis (A2P2 vs. non A2P2: hazard ratio 0.41 [0.60, 9.95], *p* = 0.21) (Table 4). Similarly, 1-year HFH rates were not statistically different between groups (log-rank *p* = 0.99), with both groups experiencing ∼17% HFH rate; this also remained nonsignificant after adjustment (A2P2 vs. non A2P2: HR 1.64 [0.31, 1.21], *p* = 0.16) (Figure 8B). The composite outcome of all-cause mortality/HFH was also similar between groups (A2P2: 25.2%, non-A2P2: 26.2%, log-rank *p*-value = 0.88) and was also not significant on adjustment in Cox-proportional hazards analysis (A2P2 vs. non A2P2: HR 1.31 [0.41, 1.41], *p* = 0.39) (Figure 8C).

**Table 4:** Unadjusted and adjusted cox regression models

	Unadjusted		Adjusted	
	Hazard ratio (95% CI)	*p* value	Hazard ratio (95% CI)	*p* value
All-cause mortality	0.77 (0.64, 2.63)	0.47	0.41 (0.60, 9.95)	0.21
HFH	1.00 (0.58, 1.75)	0.99	1.64 (0.31, 1.21)	0.16
Composite of mortality or HFH	1.04 (0.62, 1.51)	0.88	1.31 (0.41, 1.41)	0.39

Adjusted hazard ratios accounted for the following baseline characteristics: LVEF, LVESV, LVEDV, LVESD, LVEDD, etiology of secondary MR, prior MR procedure, renal failure and prior HFH.

Abbreviations: HFH = heart failure hospitalization, LVEF = LV ejection fraction, LVESV = LV end-systolic volume, LVEDV = LV end-diastolic volume, LVESD = LV end-systolic dimension, LVEDD = LV end-diastolic dimension, MR = mitral regurgitation.

## Discussion

In this pooled analysis of the EXPAND and EXPAND G4 studies, we evaluated the impact of main MR jet location—central (A2P2) versus noncentral (A1/P1 or A3/P3)—on procedural success and clinical outcomes following M-TEER with the MitraClip system. This report currently represents the largest published analysis, encompassing 1866 patients with ECL-adjudicated main MR jet location. Most patients had A2P2 jets (95.6%), whereas a smaller subset (4.3%) exhibited non-A2P2 jets. Despite anatomical differences, procedural success was high and comparable between groups (95.9% A2P2 vs. 92.5% non-A2P2). Rates of MAEs, HFH, and all-cause mortality at 1 year were similar. Durable MR reduction, sustained improvement in NYHA functional class, and significant quality-of-life benefit were observed irrespective of the main MR jet location. These results suggest that anatomies with non-A2P2 jets, once considered a technical limitation, do not significantly compromise the safety or efficacy of contemporary MitraClip therapy.

Historically, M-TEER was developed and validated in anatomies with central A2P2 pathology, as exemplified by the early EVEREST (Endovascular Valve Edge-to-Edge Repair Study) trials, which excluded patients with noncentral pathology.^2^ These early trials focused on central A2P2 MR jets to allow for a controlled learning curve since central jets are relatively free of chordae tendineae and device steering and positioning can be more straightforward in the center of the left atrium. Although early registry data raised concerns about procedural complexity and durability in noncentral lesions due to challenging chordal anatomy and variable coaptation planes, subsequent experience has led to refined procedural techniques for the treatment of noncentral MR. Consistent procedural success is achievable with careful attention to transseptal puncture height and location, which enables optimal positioning over medial or lateral jets.^9^ Additional procedural refinements, such as precise clip alignment before crossing into the left ventricle and minimizing clip movement beneath the leaflets, have helped mitigate the risk of chordal entanglement and grasping interference. These improvements are reflected in similar procedural times and low rates of SLDA between central and noncentral MR cases.

Multiple smaller prior studies have demonstrated feasibility and favorable outcomes in noncentral MR, although most of these reports focused on patients with primary (degenerative) MR.^10^^,^^11^ A unique aspect of our study, beyond its large sample size, is the inclusion of patients with secondary (functional) noncentral MR. Although the majority of noncentral MR cases have degenerative pathology involving the medial or lateral segments, our pooled analysis shows that noncentral pathology can also occur in SMR, and that these patients can be treated successfully, with clinical outcomes equivalent to those with central SMR. The underlying pathophysiology that leads to noncentral SMR is likely related to asymmetric tethering of the MV leaflets due to enlarged left ventricular dimensions.

One of the first reports on treating noncentral MR^10^ included 79 patients with PMR treated with the MitraClip system across 3 experienced European centers. Of these, 49 patients (62%) had central MR, whereas 30 patients (38%) had noncentral MR. Patients in the noncentral group exhibited wider preprocedural vena contracta and higher pulmonary artery systolic pressures compared with those in the central group, suggesting a greater baseline hemodynamic burden. Despite these differences, procedural success rates were nearly identical (95.5% central vs. 96.7% noncentral), and improvements in MR reduction, NYHA functional class, and event-free survival were observed in both groups.^10^ Similarly, Wei et al.^11^ assessed 136 patients with degenerative MR treated in China between 2021 and 2024. Seventy-seven patients were categorized with central lesions, whereas 59 were categorized as noncentral. Baseline demographics were broadly comparable, although patients with noncentral lesions tended to have better left ventricular systolic function (e.g., higher LVEF). Procedural success was again equivalent (93.5% central vs. 91.5% noncentral), with durable MR reduction achieved in both groups at discharge. Over 3 years of follow-up, recurrence-free survival rates were similar (90.3% central vs. 94.9% noncentral). Multivariable analysis identified higher postprocedural MV gradient and a leaflet-to-annulus index ≤1.2 as predictors of recurrence, but jet location was not an independent risk factor.^11^

Most recently, the EXPAND and EXPAND G4 studies provide additional evidence in broader patient populations.^5^^,^^6^^,^^12^ These studies specifically enrolled patients with a wide range of anatomical complexities, including those who would have been considered unsuitable by earlier EVEREST criteria.^2^ Anatomical complexity in these studies was defined by features such as commissural or paracommissural jets, multisegment prolapse or flail, calcification in the grasping zone, small valve areas at risk for stenosis, and prior mitral interventions. Importantly, these cohorts explicitly included patients with nonprimary central pathology, demonstrating that the latest device iterations (G3 and G4) with wider clip arms and independent leaflet grasping could reliably treat anatomies once considered unfavorable.^13^ Our analysis suggests that M-TEER operators selected specific clip types to match the anatomy of the valve anatomy.

Specifically, there was a higher utilization of narrow TEER devices in the non-A2P2 group, which may allow easier navigation in dense chordal anatomy (narrow NT + XT use in A2P2 11.5% vs. non-A2P2 27.1%). The use of nonwide clips, such as XT and NT, can be useful in noncentral pathology where there is an increased chordal density and increased risk of chordal interaction. Other considerations for the use of narrow clips are in patients with a reduced MV area at risk of stenosis, as the use of a narrow clips can minimize the risk of subsequent mitral stenosis. In addition, shorter-armed TEER devices were used more commonly in the non-A2P2 group, which more closely match the shorter leaflet lengths in these locations (NT + NTW use in A2P2 34.1% vs. non-A2P2 45.9%). In addition, non-A2P2 pathology frequently includes commissural lesions, which are often more technically complex due to the dense fan-shaped chordae and the variable coaptation planes near the commissures. Several reports have demonstrated the feasibility of treating commissural mitral regurgitation with the MitraClip System.^14^, ^15^, ^16^ Early surgical experience with edge-to-edge repair also highlighted the reproducibility of commissural approximation techniques. Alfieri et al.^17^ described that the edge-to-edge technique can be applied successfully to noncentral MR and noted that “commissural edge-to-edge repair with annuloplasty is probably the simplest and most reproducible method to repair commissural lesions.” It should be noted, however, that our current study does not provide lesion-level granularity to distinguish commissural lesions from other noncentral pathologies (e.g., lateral or medial scallops), and further research is needed to specifically characterize outcomes in this subgroup.

Our analysis provides the largest data set to date focused on main MR jet location. Compared with earlier single-center registries and smaller multicenter cohorts, we show that procedural success and 1-year clinical outcomes remain robust in non-A2P2 MR, even in a real-world, multinational cohort. Although the non-A2P2 group demonstrated slightly lower rates of MR ≤1+ at 1 year (84 vs. 91%), this did not translate into differences in mortality, rehospitalization, or quality-of-life improvement. These results align with Wei et al.^11^ and Estevez-Loureiro et al.,^10^ both in which reported comparable mid-term outcomes between central and noncentral lesions.

Importantly, the higher proportion of degenerative MR and prior mitral interventions among non-A2P2 patients in our cohort underscores that this subgroup may represent a distinct and often more complex clinical population. Nevertheless, the favorable outcomes observed highlight that non-A2P2 MR should not be considered a contraindication to M-TEER in the modern era.

### Limitations

Enrollment into the EXPAND and EXPAND G4 was conducted per site evaluation and reflects regional differences in echocardiographic guidelines. The current study did not distinguish between noncentral MR location and commissural MR. The EXPANDed studies are postmarket observational studies, which lack a control arm or blinding. Finally, all analysis was performed post hoc; therefore, the number of patients in the analysis subgroups are limited due to data availability for the subgroup definitions and study groups were imbalanced in sample size, which severely limit the statistical power, particularly for the lower-incidence outcomes described here.

## Conclusions

The findings of this analysis reinforce the expanding role of M-TEER across the spectrum of mitral anatomies. With the advent of the fourth-generation MitraClip devices and growing operator expertise, concerns regarding anatomical suitability for M-TEER should be reconsidered. Patients with non-central MR—once excluded from pivotal trials—can achieve outcomes comparable to central lesions, supporting guideline-directed use of M-TEER in this broader group. These results also provide reassurance for heart teams in recommending M-TEER for patients with anatomically challenging, but clinically appropriate MR presentations.

## Ethics Statement

Both studies were sponsored by Abbott and conducted in accordance with the principles of the Declaration of Helsinki and Good Clinical Practice guidelines. Institutional review board or ethics committee approval was obtained at each participating site, and all patients provided written informed consent before enrollment. Additional details regarding study design and conduct have been previously published.^6^

## Funding

The EXPAND (NCT03502811) and EXPAND G4 (NCT04177394) studies were sponsored by 10.13039/100000046Abbott.

## Disclosure Statement

Jason H. Rogers is a consultant to Abbott Structural Heart, Biosense Webster, and Boston Scientific. Matthew J. Price has received consulting fees and honoraria from Abbott, Boston Scientific, InnovHeart, Medtronic, Philips Medical, W.L. Gore & Associates, and Shockwave Medical. Gagan D. Singh has received consulting fees and honoraria from Abbott, Boston Scientific, InnovHeart, Medtronic, Philips Medical, W.L. Gore & Associates, and Schockwave Medical. Paul Mahoney is a consultant and proctor for Medtronic, Edwards Lifesciences, and Boston Scientific; is a consultant for Abbott; and has been awarded research grants from Edwards Lifesciences, Medtronic Abbott, and Boston Scientific. Paolo Denti has received speaker honoraria from Abbott and Edwards Lifesciences; and has been a consultant to InnovHeart, Artiness, and Pi-Cardia. Anita Asgar is a consultant to Abbott Structural Heart, Medtronic, Edwards LifeSciences, W.L. Gore, and Anteris Technologies. Janani Aiyer and Rong Huang are employees of the sponsor, Abbott. Jose Luis Zamorano has received speaker honoraria from Pfizer, Amgen, and Daiichi Sankyo; and research grants from Abbott and Edwards Lifesciences. Dr Asch’s work as an academic core laboratory director is performed through institutional research grants (MedStar Health) with Abbott, Boston Scientific, Medtronic, Edwards Lifesciences, Neovasc, Ancora Heart, LivaNova, MVRx, InnovHeart, Polares Medical, and Aria CV. Francesco Maisano has received grants and/or institutional research support from Abbott, Medtronic, Edwards Lifesciences, Biotronik, Boston Scientific, NVT, and Terumo; has received honoraria and consulting fees (personal and institutional) from Abbott, Medtronic, Edwards Lifesciences, Xeltis, and Cardiovalve; has received royalty income and intellectual property rights from Edwards Lifesciences; and is a shareholder (including stock options) of CardioGard, Magenta, SwissVortex, Transseptal Solutions, Occlufit, 4Tech, and Perifect. Saibal Kar has received grants and institutional research support from Abbott, Boston Scientific, and Edwards Lifesciences; has received consulting fees/honoraria from Abbott, Boston Scientific, W.L. Gore, and Medtronic; served as a steering committee member of the TRILUMINATE study (Abbott) and as national principal investigator of the EXPAND study and the REPAIR MR study for Abbott. Ralph Stephan von Bardeleben has performed nonpaid trial activities for Abbott, Edwards Lifesciences, Medtronic, and the University of Göttingen (IIT); and serves as an advisory board or speaker bureau member for Abbott Cardiovascular Edwards Lifesciences, Medtronic, and NeoChord. Evelio Rodriguez has been awarded grants and support for research from Abbott, Edwards Lifesciences, Boston Scientific, AtriCure, and CardioMech; and has received honoraria or consulting fees from Abbott, Edwards Lifesciences, Philips, Teleflex, and CardioMech.

The other authors had no conflicts to declare.
